# Expression of components of the urothelial cholinergic system in bladder and cultivated primary urothelial cells of the pig

**DOI:** 10.1186/s12894-019-0495-z

**Published:** 2019-07-09

**Authors:** Dorothea Leonhäuser, Jasmin Kranz, Regina Leidolf, Patrick Arndt, Ulrich Schwantes, Joachim Geyer, Joachim O. Grosse

**Affiliations:** 1 Department of Urology, RWTH Aachen University Hospital, Pauwelsstraße 30, 52074 Aachen, Germany; 2 Institute of Pharmacology and Toxicology, Biomedical Research Center BFS, Justus Liebig University Giessen, Giessen, Germany; 3 Dr. R. Pfleger GmbH, Bamberg, Germany

**Keywords:** Urothelium, Urinary bladder, Acetylcholine, Transporter, Muscarinic receptor, German landrace pig, Göttingen Minipig

## Abstract

**Background:**

Porcine urinary bladders are widely used for uro-pharmacological examinations due to their resemblance to the human organ. However, characterisations of the porcine urothelium at the molecular level are scarce up to now. As it has become clear over the last years that this tissue plays an important role in the signaling-pathways of the bladder, we examined whether the transporter and receptor pattern (with focus on the transmitter acetylcholine) is comparable to the human urothelium. With regard to in vitro *studies*, we also investigated if there is a difference between the native tissue and cultivated primary urothelial cells in culture.

**Methods:**

Urothelium from German Landrace and Göttingen Minipig bladders was collected. One part of the German Landrace tissue was used for cultivation, and different passages of the urothelial cells were collected. The actual mRNA expression of different transporters and receptors was examined via quantitative real-time PCR. These included the vesicular acetylcholine transporter (VAChT), the choline acetyl transferase (ChAT), organic cation transporters 1–3 (OCT1–3), organic anion transporting polypeptide 1A2 (OATP1A2), P-glycoprotein (ABCB1), the carnitine acetyl-transferase (CarAT), as well as the muscarinic receptors 1–5 (M1–5).

**Results:**

There is a strong qualitative resemblance between the human and the porcine urothelium with regard to the investigated cholinergic receptors, enzymes and transporters. CarAT, OCT1–3, OATP1A2 and ABCB1 could be detected in the urothelium of both pig races. Moreover, all 5 M-receptors were prominent with an emphasis on M2 and M3. VAChT and ChAT could not be detected at all. Cultures of the derived urothelial cells showed decreased expression of all targets apart from ABCB1 and CarAT.

**Conclusions:**

Based on the expression pattern of receptors, transporters and enzymes of the cholinergic system, the porcine urinary bladder can be regarded as a good model for pharmacological studies. However, cultivation of primary urothelial cells resulted in a significant drop in mRNA expression of the targets. Therefore, it can be concluded that the intact porcine urothelium, or the whole pig bladder, may be appropriate models for studies with anticholinergic drugs, whereas cultivated urothelial cells have some limitation due to significant changes in the expression levels of relevant targets.

## Background

Over the last years, the use of urinary pig bladders, and the pig itself as a large animal model, have become extremely popular for urological examinations. Especially pharmacological studies depend on the testing of drugs using porcine material [1–3]. This is justified on the basis that the porcine bladder closely resembles the human organ both anatomically and physiologically [4, 5]. Nevertheless, not much research on the molecular basics has as yet been conducted which justifies unlimited use and comparison of the porcine material [5].

Some molecular examinations of detrusor and urothelial tissue have been performed on human material concerning transporters and receptors of anticholinergic drugs, such as trospium chloride, which are used as the clinical standard in the treatment of overactive bladder (OAB) [6–9]. Although it is clear that anticholinergic drugs block muscarinic (M-) acetylcholine (ACh) receptors, it is not completely understood how these drugs interact with the cellular machinery of the urothelium and the detrusor. Goepel et al. [10], as well as Sellers et al. [11], showed that muscarinic receptors M2 and M3 are prominent in porcine as well as human detrusor tissue, and in the human urothelium all five known M-receptor subtypes are present [12–14].

Besides the M-receptors, other possible drug targets exist in the human bladder. Lips et al. [15] found proof for different transporters and enzymes responsible for synthesis and storage of ACh in the murine and human urothelium, including the carnitine acetyl-transferase (CarAT), whereas the classical ACh synthesising enzyme of neuronal cells, choline acetyltransferase (ChAT), seems to be absent in the urothelium. Furthermore, they demonstrated expression of the organic cation transporters OCT1–3 in the human urothelium. Interestingly, all of them are active in transporting TrCL [16]. Moreover, the solute carrier organic anion transporting polypeptide 1A2 (OATP1A2), involved in the cellular uptake of TrCL, as well as P-glycoprotein (syn. ATP-binding cassette transporter ABCB1, encoded by the multi-drug resistance gene MDR1), involved in the efflux of TrCL, are expressed in the normal urothelium [17–19]. The vesicular ACh transporter VAChT, which transports ACh in synaptic vesicles of neuronal cells, appears to be absent in the urothelium [13].

With regard to the pig as a model for pharmacological studies on anticholinergic drugs in the bladder, our aim was to determine whether these relevant transporters, enzymes and receptors are present in the porcine urothelium with the same expression pattern and to the same extent as in the human. Furthermore, their molecular stability during cell culture was to be examined.

## Methods

### Harvest and storage of urothelial tissue and cell culture of urothelial cells (UC)

All experiments on animals were performed in compliance with German legislation governing animal studies and the Guide for the Care and Use of Laboratory Animals (National Institutes of Health (NIH), Publication No 85–23, revised 2011). The pig urinary bladders were obtained from other working groups in our animal facility in order to reduce the number of animal experiments (3-R principle). These animal experiments were approved by the Governmental Animal Care and Use Committee (LANUV Recklinghausen). Göttingen Minipigs (GM) were about 2 years old whereas the German Landrace (GL) pigs were 6 months old. Besides the anaesthetics, no other drugs or treatments were applied to the pigs which could affect the bladder tissue. The pigs were euthanized by the original working group using 0.16 g/kg barbiturate i.v. (Narcoren®, Merial, Hallbergmoos, Germany), and the intact bladders were transported to the cell culture facility in a 37 °C pre-warmed Modified Eagle’s Medium (MEM, Life Technologies, Braunschweig, Germany).

Via a Y-shaped incision, bladders from GM and GL were opened, and the urothelial tissue was carefully dissected and minced with scissors. Whole urothelial tissue of GM and one segment of GL tissue was frozen in liquid nitrogen and stored at − 80 °C. The other segment of GL tissue was used for cell culture as described previously [20]. Briefly, the urothelium was incubated in MEM containing 400 μg/ml collagenase (Liberase®, Roche Applied Sciences, Penzberg, Germany) for 1 h at 37 °C. The cell suspension was then filtered, washed with MEM containing 10% fetal calf serum (FCS, Thermo Scientific) and transferred into collagen-coated (Biochrom AG, Berlin, Germany) cell culture flasks (Nunclon™, Thermo Scientific). Incubation of the urothelial cells (UC) for the first 24 h was performed in MEM containing 20% FCS, 1% gentamicin (PAA, GE Healthcare, Frankfurt am Main, Germany) and 1% amphotericin B (PAA). After 24 h, the medium was changed to Keratinocyte-SFM (Life Technologies) and subsequently changed twice a week. Examination of the cells in culture was performed with a Leica DMI 4000B (Leica Microsystems GmbH, Wetzlar, Germany) with integrated software Diskus (4.80.5909, Hilgers, Technisches Büro, Königswinter, Germany). The cells were split at confluence and 1 × 10^6^ cells were transferred into a new 75 cm^2^ flask. The remaining cells were frozen in liquid nitrogen and stored at − 80 °C.

### Immunohistochemical staining of tissue and cells

Validation of the cell type was performed via immunohistochemistry. Therefore, confluent UC were detached with trypsin-EDTA (Life Technologies), washed with phosphate buffered saline (PBS) (Life Technologies) and fixed in 4% (w/v) phosphate buffered formaldehyde (Merck). After further centrifugation, supernatant formaldehyde was removed and the cells were mixed with 3% (w/v) agarose (Biozym Scientific GmbH, Hessisch Oldendorf, Germany). The agarose cell-hybrids were cooled for 3 min. in a fridge at 4 °C. Native bladder tissue served as the control and was also used for staining of muscarinic receptors M2 and M3 and therefore fixed in phosphate buffered formaldehyde. The cell-hybrids and the native tissue were dehydrated, embedded in paraffin and cut into 3 μm sections.

Antigen retrieval of deparaffinised sections was performed using citrate-buffer (Zytomed Systems GmbH, Berlin, Germany) in a steamer for 30 min. Primary monoclonal and polyclonal antibodies (Table 1) were incubated for 1 h, and secondary antibody and chromogen development (DAKO Real EnVision HRP rabbit/mouse with DAB) were applied according to the manufacturer’s protocol. Counterstaining was performed using haemalaun (Merck). Staining was observed using a Leica DM6000B and integrated software *Diskus* (4.80.5909, Hilgers, Technisches Büro, Königswinter, Germany).

**Table 1:** Primary antibodies for immunohistochemistry

Antibody	Clone number	Reactivity	Dilution	Incubation time	Company
PanCK	AE1/AE3	Monoclonal mouse, anti-human	1:300	1 h	Dako GmbH, Hamburg, Germany
Anti-Muscarinic Acetylcholine Receptor 2	31-1D1	Monoclonal mouse, anti-human	1:100	1 h	Abcam, Cambridge, United Kingdom
Anti-Muscarinic Acetylcholine Receptor M3 antibody	–	Polyclonal rabbit, anti-pig	1:100	1 h	Abcam, Cambridge, United Kingdom

### Quantitative real-time polymerase chain reaction

TriReagent (Sigma Aldrich) was used to extract RNA from cultured cells and tissues according to the manufacturer’s protocol. The isolated RNA was reverse-transcribed using the SuperScript III system (Life technologies GmbH, Darmstadt, Germany). Quantitative real-time polymerase chain reaction (qRT-PCR) was performed using the TaqMan GEX Master Mix (Life Technologies). Four of the obtained assays (VAChT, OCT3, CHRM4, and CHRM5) had to be custom-made as there were no such probes available for the pig. All other TaqMan assays were ordered according to the reference numbers shown in Table 2. Glyercinaldehyde-3-phosphate-dehydrogenase (GAPDH) was used as the housekeeping gene. All assays were tested using a pig tissue cDNA panel (BioCat GmbH, Heidelberg, Germany) prior to the initial examinations.

**Table 2:** TaqMan gene expression assays used for real-time PCR expression analysis

Assay	Order No.
VAChT-Pig	Custom Gene ex assay, AIWR3FN, referred to the cDNA sequence with GenBank Accession No. XM_013983446
ChAT-Pig	Taqman Gene ex assay MTO, sm, Ss03391504_m1
CarAT-Pig	Taqman Gene ex assay MTO, sm, Ss03389781_m1
ABCB1-Pig	Taqman Gene ex assay MTO, sm, Ss03373435_m1
OCT1-Pig	Taqman Gene ex assay MTO, sm, Ss03391173_m1
OCT2-Pig	Taqman Gene ex assay MTO, sm, Ss03390935
OCT3-Pig	Custom Gene ex assay, JD89KCA, referred to the cDNA sequence with GenBank Accession No. XM_003121106
OATP1A2-Pig	Taqman Gene ex assay MTO, sm, Ss03375623_u1
CHRM1-Pig	Taqman Gene ex assay MTO, sm, Ss03393581_u1
CHRM2-Pig	Taqman Gene ex assay MTO, sm, Ss03383697_u1
CHRM3-Pig	Taqman Gene ex assay MTO, sm, Ss03387661_u1
CHRM4-Pig	Custom plus Tqmn RNA Assays, AJPADK0, referred to the cDNA sequence with GenBank Accession No. XM_003122828
CHRM5-Pig	Custom plus Tqmn RNA Assays, AJN1FES, referred to the cDNA sequence with GenBank Accession No. XM_021099908
GAPDH-Pig	FG, Off the shelf GX Set, Ss03374854_g1

### Statistical analysis

Cell cultures from three different animals were examined using technical triplicates. Statistical evaluation was performed using OriginPro (2017G, Origin Lab Corporation, Northampton, USA). The Shapiro-Wilks test was used to test for normal distribution, and One-Way ANOVA with the Tukey-Post Hoc test was used to determine statistical differences. Values of *p* ≤ 0.05 were considered to be significant.

## Results

### Cell culture of urothelial cells and proof of cell type

**Fig. 1:**
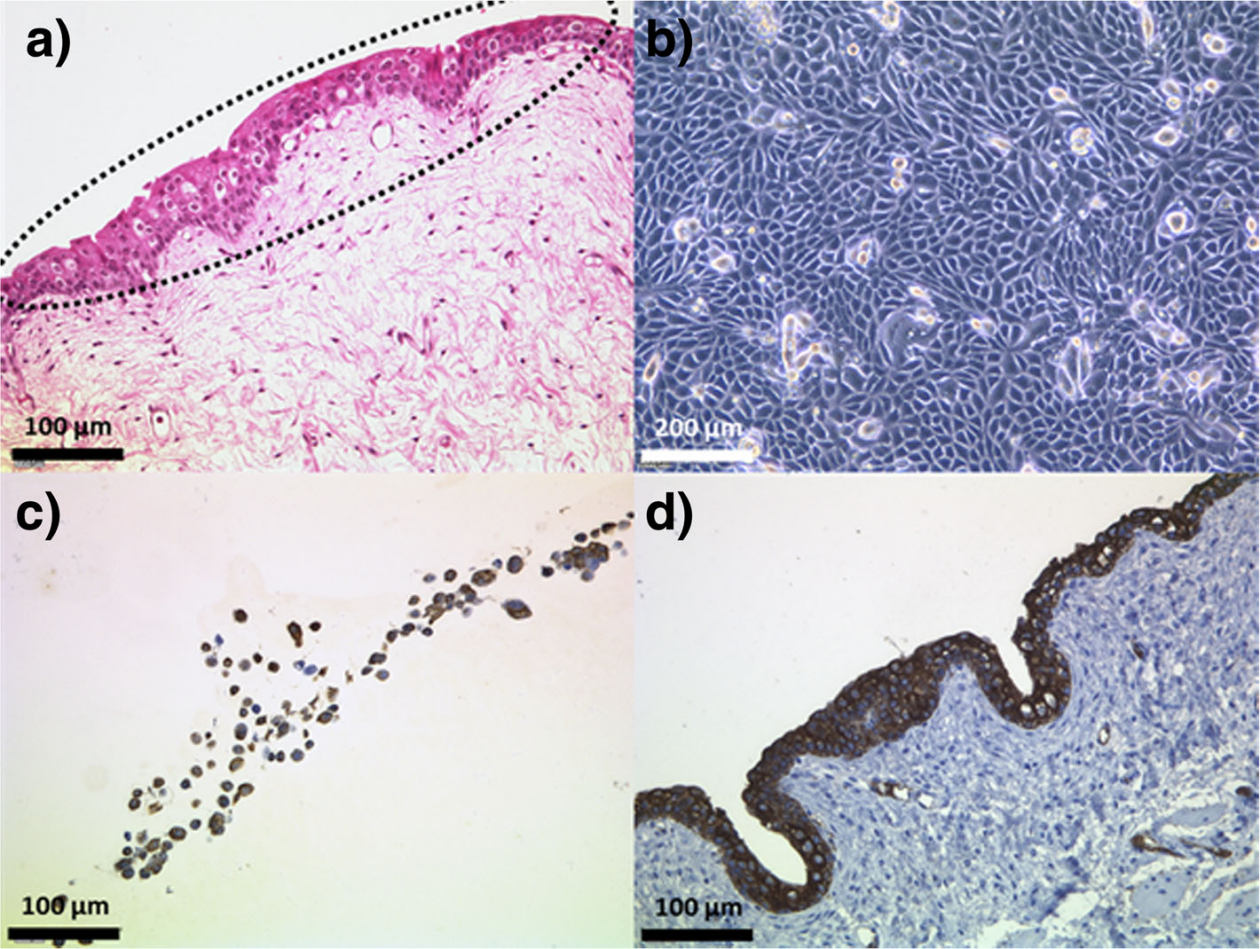
Isolation of urothelial cells from German Landrace pig urothelium. **a** Urothelial layer which was dissected from the underlying lamina propria (HE staining). **b** Urothelial cells in culture at confluence. **c** Immunostaining of UCs with panCK embedded in agarose. **d** Positive control of porcine urothelium, immunostained with panCK

**Fig. 2:**
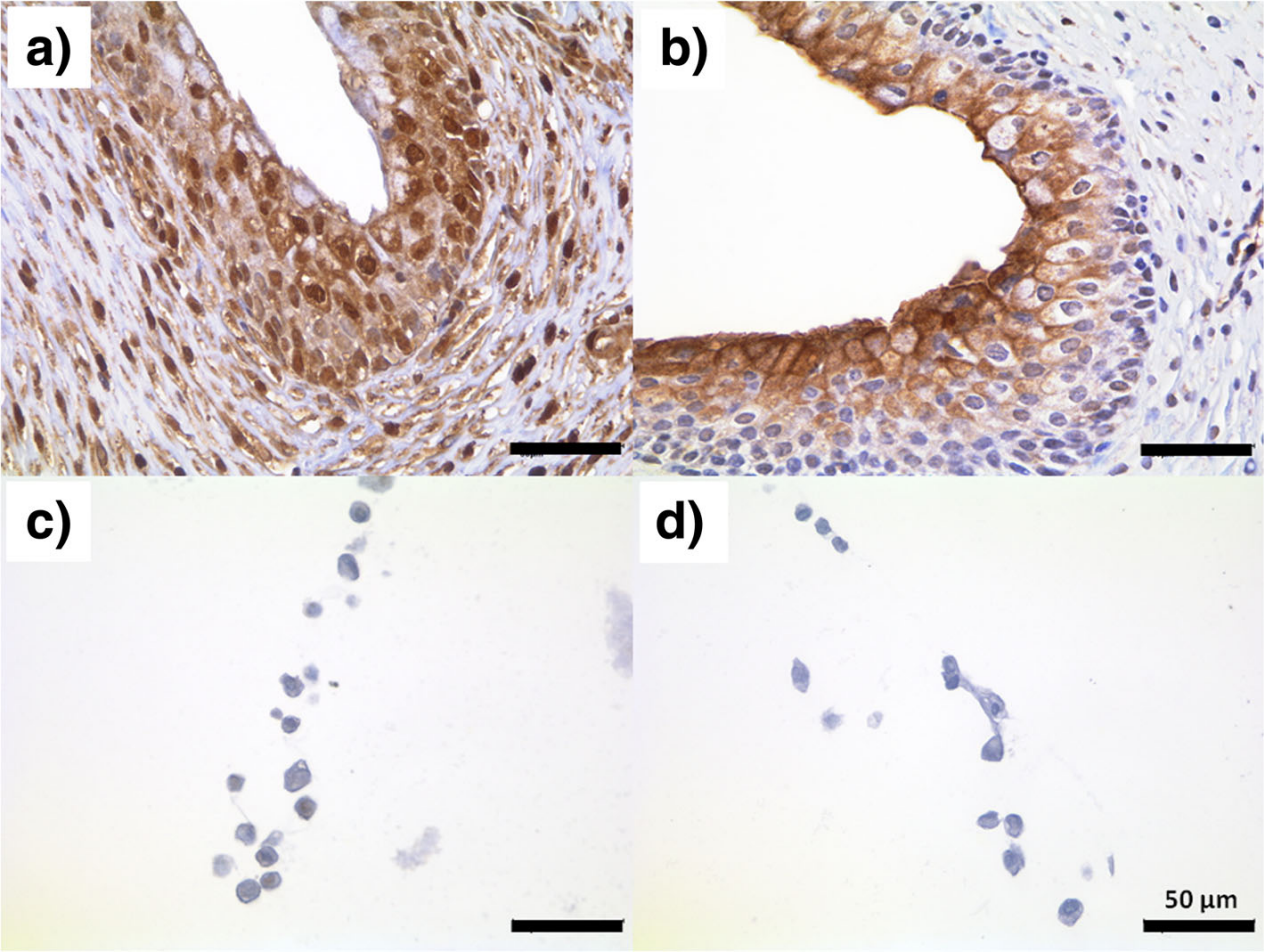
Immunohistochemical staining of muscarinic receptors in native bladder tissue and derived urothelial cells. **a**+**c** Muscarinic receptor M2 and **b**+**d** M3 could be visualized in **a**+**b** native bladder tissue of the German Landrace pig but **c**+**d** not in the derived urothelial cells at passage 1. Scale bar = 50 μm

During the cultivation period, UCs from GL could be passaged up to three times. This resulted in five samples per GL pig; the untreated bladder biopsy and four immediately consecutive passages of UCs. From these GL pig bladders and three additional GM bladders, untreated urothelial tissue samples were frozen to compare the native tissues of the different races. Immunohistochemical staining of the cells against panCK confirmed an urothelial phenotype (Fig. 1). The presence of muscarinic receptors M2 and M3, most relevant for storing and voiding of urine in the bladder, could also be visualized in the native urothelium of the German Landrace pig (Fig. 2a+b).

### Real-time PCR

**Fig. 3:**
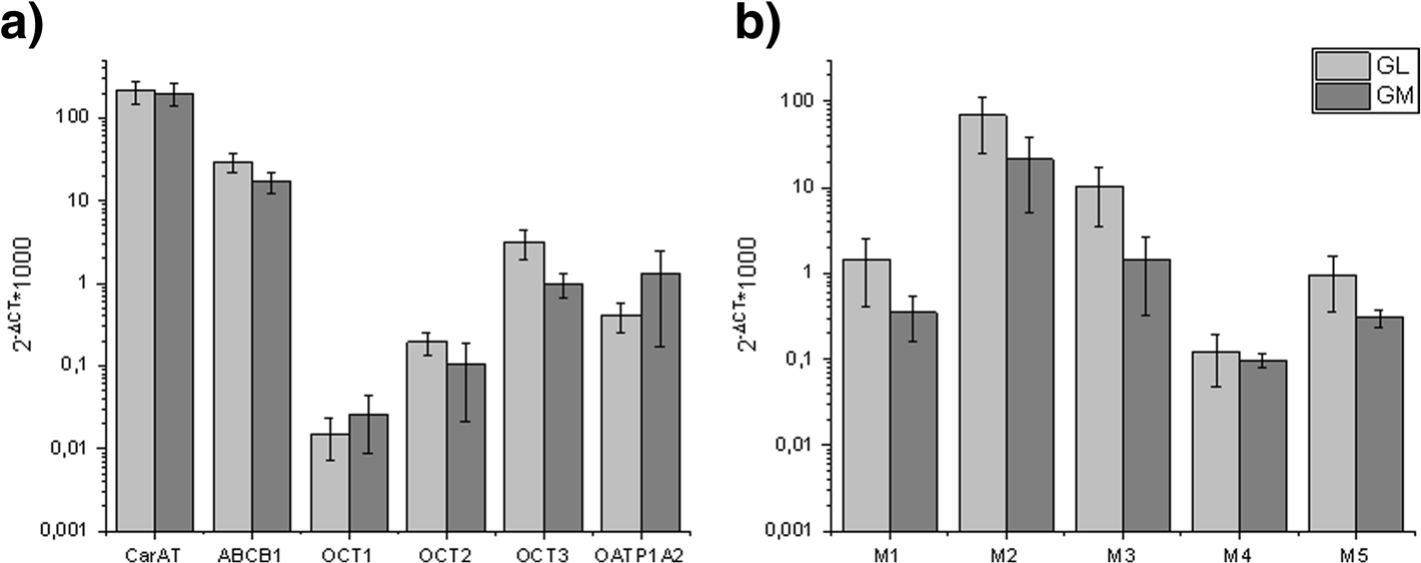
Relative gene expression in the urothelium of German Landrace pigs and Göttingen minipigs (*n* = 3). VAChT and ChAT expression could not be detected. All enzymes/transporters (**a**) as well as receptors (**b**) relevant for anticholinergic therapy showed comparable expression pattern for GL and GM. Highest expression was detected for CarAT, ABCB1, OCT3, OATP1A2, M2 and M3. GAPDH expression was used for normalization

In general, quantitative real-time PCR expression analysis of native porcine urothelium was very similar concerning the presence of the examined transporters and receptors as in humans [13, 15]. The enzyme CarAT and the transporter ABCB1 were most commonly expressed. The organic cation transporters OCT1–3, as well as OATP1A2, could be detected, but to a lesser extent (Fig. 3a). The mRNA expression for all muscarinic receptors was found in the porcine urothelium, especially M2 and M3 (Fig. 3b). VaChT and ChAT, as in human urothelium, were not detectable. There were no significant differences in the expression pattern between the two pig breeds.

**Fig. 4:**
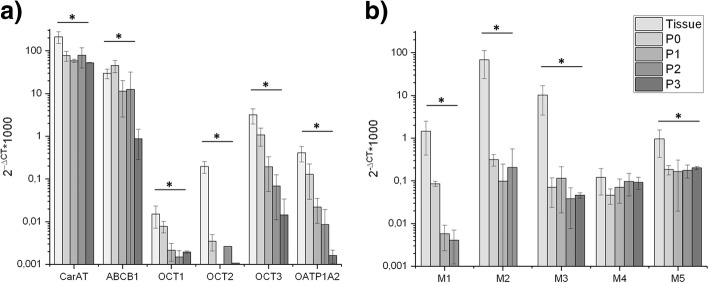
Relative gene expression in the urothelium from German Landrace pigs (“Tissue”) as well as derived UCs (“P0-P3”) (*n* = 3). All enzymes/transporters (**a**) as well as receptors (**b**) relevant for anticholinergic therapy were down-regulated during repeated passaging, with the exception of M4. In particular, expression levels of OCT2 and M1 dramatically dropped to nearly undetectable levels. GAPDH expression was used for normalization. * Significant down-regulation compared to tissue with *p* < 0.05

Subsequently, it was analysed whether the enzyme/transporter/receptor expression changed during cultivation of the porcine urothelium. In general, most of the analysed targets were down-regulated following repeated passaging of the cells. This down-regulation was most noticeable for OCT1 and OCT2 as well as M1-M3, whereas the expression level of the M4 and M5 receptors was almost maintained up to passage 3 (P3) (Fig. 4). The downregulation of muscarinic receptors M2 and M3 could also be visualized by immunohistochemical staining (Fig. 2c+d).

## Discussion

Previously, systematic examinations on the urothelium as an active component of the bladder storage and micturition process have been overlooked. This might be due to the fact, that this special tissue was only assumed to be a barrier with no physiological impact on the bladder function [21, 22]. As it has become increasingly clear that the urothelium plays an essential role, it is even more important to re-examine the animal models already used for translational examinations applicable to the human bladder.

So far, we have only established the M2 and M3 immunohistostaining for the targets also analysed via qRT-PCR. As it is not easy to find antibodies that are applicable for pig tissue, it is often necessary to use antibodies for human or other species and hope for cross-reactions. Therefore, we also tried a GAPDH antibody which turned out to react very unspecific. As the use of immune staining is more for visual effects, we postponed the establishment of other antibodies.

Using qRT-PCR expression analysis of selected enzymes, transporters and receptors of the urothelial cholinergic system, a comparable expression pattern was detected in the pig in the present study, as has previously been described for humans [13, 15, 18, 19]. Although VAChT is an important vesicular transporter for ACh, it could not be detected in the porcine urothelium. This too, is in accordance with the findings in the human urothelium, where the presence of VAChT could not be proved [15]. However, as a limitation it has to be mentioned that the gene expression assay for VAChT was derived from a predicted cDNA sequence (see Table 1) and could not be confirmed in any porcine tissue so far. Additionally, in the present study, expression of the classical ACh synthesising enzyme ChAT could not be detected in the porcine urothelium as has previously been reported for the human [13]. However, the porcine urothelium showed high mRNA expression levels for CarAT, which is an alternative source of ACh synthesis in the urothelium [13]. In the pig, all three OCTs (OCT1–3) were detected in the urothelium in the order OCT3 > OCT2 > OCT1, and all of them were also present in the human urothelium [13]. The solute carrier OATP1A2, uptake carrier for endogenous substances and pharmaceuticals in the human urothelium, has been identified as a transporter for TrCL by Bexten et al. [19], and could also be detected in the pig urothelium by our group. ABCB1 plays an important role in the efflux of many drugs, also including the anticholinergic drug TrCL [23]. ABCB1 is highly expressed in normal human urothelium [18, 19] and the present study also confirmed its expression in the porcine urothelium. Finally, all 5 M-receptors have been reported in the human urothelium [13, 14], and could be detected in the present study with a similar expression pattern in the pig, with the highest expression levels for M2 and M3.

Two different pig races were analysed i.e. GL and GM, and both showed comparable expression patterns to each other and the human. Taking this into account, it could be expected that pharmacological studies in pigs, for example with anticholinergic drugs, would substantially reflect the situation in man.

Cultivation of urothelial cells has previously been established in order to avoid repeated in vivo experiments in pigs, and to enable in vitro studies with urothelial derived cells. For this reason, the expression of the relevant enzymes, transporters, and receptors was also analysed under cell culture conditions during repeated passaging. Unfortunately, most of the analysed targets revealed continuous downregulation over time, which in general limits the usability of these cells, for example, for transport or receptor binding experiments at higher passaging frequencies. Bexten et al. were able to show that, on the one hand, TrCL is a substrate of the aforementioned solute carriers OCT1 and OATP1A2 (uptake), but on the other hand also for the efflux carrier ABCB1 [19]. The in vitro downregulation of the uptake transporters in combination with the still relatively high amount of the ABCB1 efflux carrier would lead to a non-physiological shift compared to the actual in vivo situation. This cultivation-related downregulation could also be observed for the muscarinic receptors, especially M2 and M3, which play a crucial role in the storing and voiding mechanisms of the bladder. M3 is known to mediate the contractile response and thus is addressed by the main muscarinic receptor antagonist TrCL [12]. This has to be considered for uptake studies with anticholinergic drugs like TrCL. However, downregulation of M1-M5 has also been demonstrated for human urothelial cells in culture by Tyagi et al. [14].

Interestingly, ABCB1 showed slight upregulation during the first round of cultivation in comparison to the tissue samples. This upregulation of ABCB1 might be triggered by cell culture supplements such as antibiotics, as the main role of this transporter is the efflux of potential hazardous substances out of the cell [23, 24].

UCs in culture are a promising model for pharmacological in vitro studies with anticholinergic drugs. However, such studies would be disadvantaged by down-regulation of relevant transporters and receptors for ACh and / or anticholinergic drugs. Nevertheless, Mukerji et al. [25], as well as Gupta et al. [26], could show that urothelial cells of patients with interstitial cystitis (IC) retained their phenotype under cell culture conditions. Furthermore, the animals in this study were mature but not old [27, 28] and thus, do not represent the aged population which suffer from bladder dysfunctions. Therefore, a corresponding animal model with IC or OAB could provide more insight in the mechanisms of these diseases at a molecular level.

It remains that physiological and pharmacological studies on the urinary bladder are mainly conducted in rodent models [29–32]. These animals are cheap, easy to handle, and a large number of individuals can be investigated. However, data generated in rodents may not be fully comparable to the situation in humans, as the anatomy, physiology and the day-night-rhythm of these animals is somewhat different. Therefore, pigs were analysed in the present study as these show a more comparable bladder physiology to humans [4].

Initially the study was intended only with GL pigs as these are cheaper to obtain and therefore used more commonly in animal studies. Additionally, it is common practice to perform whole bladder experiments and drug studies using pig bladders from an abattoir [1–3, 33]. However, the GM has proven itself to be a comparable animal model for urological in vivo long-term studies, not only on the physiological [4] but also on the molecular level, as was also demonstrated in the present study. Based on the data presented here, both pig races are appropriate as a pharmacological animal model concerning the investigated targets.

## Conclusions

This study was able to show that the porcine urothelium of the GL and GM pig is very similar to the human urothelium concerning the investigated ACh-dependent targets. We therefore conclude that both pig races are appropriate as a pharmacological animal model for in vivo and ex vivo investigations. Nevertheless, the use of UCs from healthy animals is limited due to the down-regulation of the afore-mentioned targets. Therefore, the development and use of an animal model with OAB or IC could provide more insight into the working mechanism of these diseases.

## Data Availability

The datasets used and/or analysed during the current study are available from the corresponding author on reasonable request.

## Abbreviations

ABCB1: ATP-binding cassette B1
ACh: Acetylcholine
CarAT: Carnitine acetyl-transferase
ChAT: Choline acetyl transferase
GAPDH: Glyercinaldehyde-3-phosphate-dehydrogenase
GL: German Landrace pig
GM: Göttingen Minipig
IC: Interstitial cystitis
M1–5: Muscarinic receptors 1–5
MEM: Modified Eagle’s Medium
OAB: Overactive bladder
OATP1A2: Organic anion transporting polypeptide 1A2
OCT1–3: Organic cation transporters 1–3
PBS: Phosphate buffered saline
TrCL: Trospium chloride
UC: Urothelial cell
VAChT: Vesicular acetylcholine transporter
